# Unilateral frosted branch angiitis in an human immunodeficiency virus-infected patient with concurrent COVID-19 infection: a case report

**DOI:** 10.1186/s13256-021-02826-1

**Published:** 2021-05-12

**Authors:** Tsu Hong Lim, Yong Zheng Wai, Jia Cherng Chong

**Affiliations:** Department of Ophthalmology, Queen Elizabeth Hospital, Sabah, Malaysia

**Keywords:** Frosted branch angiitis, Coronavirus disease 2019, Cytomegalovirus

## Abstract

**Background:**

Frosted branch angiitis (FBA) is an uncommon ocular sign with multiple causes. With the recent outbreak of coronavirus disease 2019 (COVID-19), many cases of ocular manifestation in association with this disease have been reported. However, as yet we have no complete understanding of this condition. We report here the first case of FBA in a human immunodeficiency virus-infected patient with coexisting cytomegalovirus (CMV) and COVID-19 infection.

**Case presentation:**

A 33-year-old Malay man with underlying acquired immunodeficiency syndrome receiving highly active antiretroviral therapy was referred to the Opthalmology Department with complaints of blurry vision for the past 2 months. He had tested positive for and been diagnosed with COVID-19 1 month previously. Clinical examination of the fundus revealed extensive perivascular sheathing of both the artery and vein suggestive of FBA in the right eye. Laboratory testing of nasal swabs for COVID-19 polymerase chain reaction (PCR) and serum CMV antibody were positive. The patient was then admitted to the COVID-19 ward and treated with intravenous ganciclovir.

**Conclusion:**

Clinicians should be aware of and take the necessary standard precautions for possible coexistence of COVID-19 in an immunocompromised patient presenting with blurred vision, eye redness, dry eye and foreign body sensation despite the absence of clinical features suggestive of COVID-19. Whether FBA is one of the ocular signs of co-infection of COVID-19 and CMV remains unknown. Further studies are needed to provide more information on ocular signs presented in patients with concurrent COVID-19 and CMV infections.

## Background

Frosted branch angiitis (FBA) is an uncommon condition characterized by extensive translucent retinal perivascular sheathing involving both arterioles and venules. It has been associated with several conditions, most commonly in immunocompromised individuals with cytomegalovirus (CMV) infection [1].

To our knowledge, there are no reported cases of FBA in a patient co-infected with CMV and coronavirus disease 2019 (COVID-19) to date. The sign and symptoms of the patient with FBA with co-infection of both CMV and COVID-19 remain unclear. We report here the first case of FBA in a patient with coexisting CMV and COVID-19 infection, with the only evident ocular symptom being blurry vision.

## Case presentation

A 33-year-old Malay man, unmarried, with no known past medical history, was newly diagnosed with acquired immunodeficiency syndrome (AIDS) in September 2020. He was the youngest of four children. At presentation, he reported that he previously had multiple sexual partners. He was a non-smoker and non-alcoholic who worked as a laborer and had experienced difficulties during the COVID-19 pandemic. He was not on any medications or oral supplements. Test results for other infective diseases, including hepatitis B, hepatitis C, toxoplasma, syphilis, cryptococcosis and tuberculosis, were unremarkable. CD4+ T lymphocyte count taken on September 2020 was 9 cells/mm^3^. He was subsequently started on highly active antiretroviral therapy (HAART) the following month, including oral efavirenz 600 mg, oral emtricitabine 200 mg and oral tenofovir 300 mg, all once daily. One month after starting the HAART regimen, he had contact with a COVID-19-positive person, and a nasal swab for the COVID-19 polymerase chain reaction (PCR) assay was performed. The result indicated that he was positive for COVID-19. He was not admitted to the hospital but was under home quarantine.

**Fig. 1:**
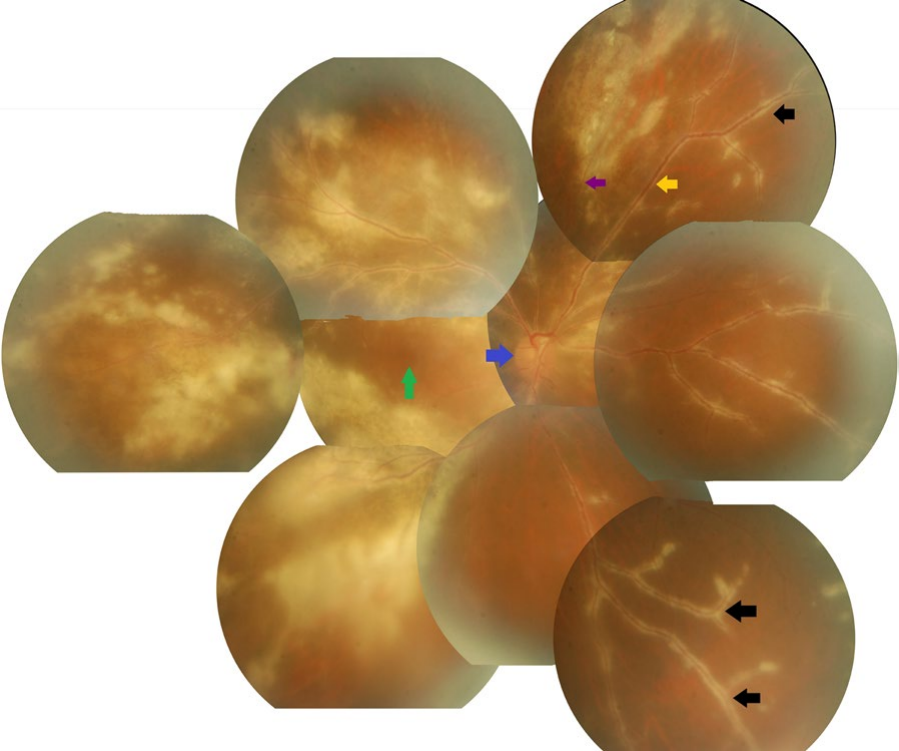
Perivascular sheathing of the artery and vein with a whitish area that surrounds the retinal vessels (black and purple arrow) suggestive of frosted branch angiitis of the right eye. The yellow and purple arrow shows the retinal vein and retinal artery, respectively. The blue arrow shows the optic disc. The green arrow shows the macula

He presented to our department in December 2020 complaining of a progressive blurring of vision in his right eye for the past 2 months, with no symptoms suggestive of COVID-19 at the time of presentation. Visual acuity in the right and left eye was 6/60 and 6/7.5, respectively. No significant abnormalities were noted upon examination of the bilateral eye anterior segment. Generalized retinal vasculitis with severe sheathing of the retinal vessels and mild vitritis was seen in the right eye on fundus examination (Fig. 1). FBA was noted. No abnormalities were found in fundus examination of the left eye. Subsequent serum serology testing for CMV was positive for both IgM and IgG. At the same time, his nasal swab for COVID-19 was still positive with a low cycle threshold value.

**Fig. 2:**
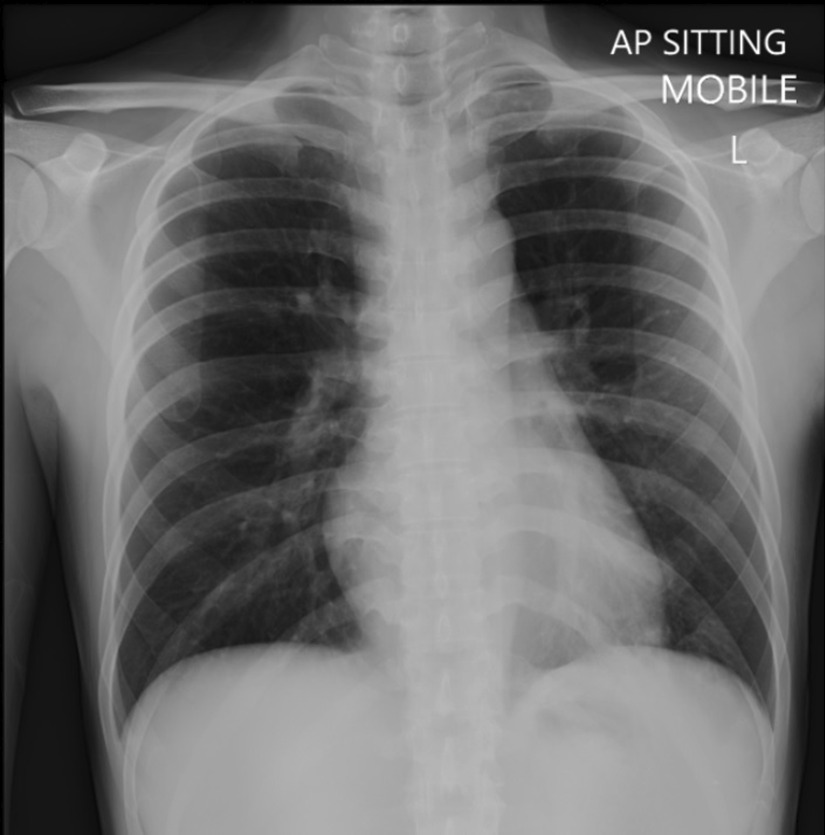
Normal chest X-ray

The patient was then admitted into the COVID-19 ward for 2 weeks. At that time, the patient had already been on HAART for 2 months. Upon admission to the COVID-19 ward, testing revealed that the CD4+ T lymphocyte count had increased from 9 to 41 cells/mm^3.^. There were no remarkable findings on physical and neurological examination upon admission to the COVID-19 ward: blood pressure was 126/85 mmHg, pulse rate was 84 beats per minute, temperature was 36.8 °C and oxygen saturation (SpO_2_) was 100% under room air. He did not have any body weakness, shortness of breath or chest pain, with clear lungs upon auscultation. Chest X-ray taken upon admission to the ward showed no significant findings, with clear lungs (Fig. 2). He was then treated with intravenous (IV) ganciclovir 225 mg twice a day (10 mg/kg/day) for 2 weeks. The patient came back to the clinic for review 1 week after the completion of his IV ganciclovir. It was noted that his FBA in his right eye had improved gradually and that his best corrected visual acuity had recovered to 6/12. Blood investigations showed a high level of acute inflammatory markers during the initial diagnosis of COVID-19, with subsequent down-trending (Table 1). The patient subsequently defaulted follow-up.

**Table 1:** Patient’s laboratory result

Parameters	Diagnosed as COVID-19 positive (21 November 2020)	Before IV ganciclovir treatment (5 December 2020)	During IV ganciclovir treatment (9 December 2020)	After IV ganciclovir treatment (20 January 2021)
Diagnosis	COVID-19 positive	FBA with COVID-19	FBA with COVID-19	Resolution of FBA
COVID-19 PCR	Positive	Positive	–	–
White cell count (10^3^/uL)	6.89	7.56	6.2	8.1
Haemoglobin (g/dl)	9.8	9.6	10.3	12.9
Platelet (10^3^/uL)	517	477	389	354
C-reactive protein (mg/L)	47	17.8	16	13
Erythrocyte sedimentation rate (mm/h)	14	16.9	22.1	2
Ferritin (ng/ml)	3985.3	1205	1070.2	474.8
Lactate Dehydrogenase (U/L)	–	301	–	221
CD4 count (cells/mm^3^)	9	41	–	–
CMV (IgM and IgG)	–	Positive	–	–
Total protein (g/L)	76	80	86	82
Albumin (g/L)	38	30	35	46
Total bilirubin (µmol/L)	5.9	3.9	3.4	6.5
Alkaline phosphate (U/L)	100	87	121	98
Alanine transaminase (U/L)	73	22	26	20
Aspartate transaminase (U/L)	60	23	24	21
Urea (mmol/L)	2.8	2.6	3.6	4.6
Creatinine (µmol/L)	58	63.9	67.7	74
Sodium (mmol/L)	136	136	133	138
Potassium (mmol/L)	4.4	4.2	4.6	4.7
Chloride (mmol/L)	101	107	105	103

*COVID-19* Coronavirus 2019,* CMV* cytomegalovirus,* FBA* frosted branch angiitis,*IV* intravenous,* PCR* polymerase chain reaction

## Discussion

We report here a case of a human immunodeficiency virus (HIV)-infected patient who presented to us 1 month after receiving a diagnosis of COVID-19, confirmed by PCR assay, with the a chief complaint of blurry vision in right eye for the past 2 months. This case is unique as, to our knowledge, there are no reported cases of a patient with co-infection of COVID-19 and CMV presenting with signs of FBA.

The term FBA was first described by Ito *et al*. back in 1976 when they found a thick perivascular sheathing in the fundus of a 6-year-old boy [2]. There are many conditions associated with FBA, some of which include Behcet’s disease, systemic lupus erythematosus, CMV retinitis, Crohn’s disease, Mycobacterium tuberculosis infection, aseptic meningitis, leukemia, lymphoma, herpes simplex type 2, varicella-zoster virus and several other bacterial and viral infections [1, 3–12]. FBA presents more commonly bilaterally, but unilateral presentation has also been reported [13].

The World Health Organization declared an international state of emergency for novel COVID-19 on 5 April 2020. The PCR assay is the current diagnostic method to diagnose COVID-19 infection, with samples taken from nasal swab, tracheal aspirate or bronchoalveolar lavage specimens [14]. However, this has to be correlated with laboratory and radiological findings, which typically show lymphocytopenia, elevated alanine transaminase and aspartate transaminase levels and increased inflammatory markers, such as C-reactive protein [14]. A computed tomography scan has a higher sensitivity to detect lung changes in COVID-19 infected patients [14], but in our case only a chest X-ray was done as computed tomography is reserved for patients with an undefined clinical picture in our hospital setting. Currently, the only drug approved by the U.S. Food and Drug Administration for the treatment of COVID-19 is the antiviral drug remdesivir. Unfortunately, our hospital does not have remdesivir; instead, favipiravir is used to treat severe symptomatic patients in our hospital. The patient was not started on favipiravir, and chest X-ray was only done once upon admission as he was classified as stage 1, which is asymptomatic, and he remained stable during his stay in the COVID-19 ward.

Ocular manifestation in a patient infected with COVID-19 is still not well established. It is suspected that viral transmission can occur through the eye and that patients can present with conjunctivitis, keratoconjunctivitis and epiphora as early symptoms of COVID-19 infection [15]. The top three COVID-19-related ocular symptoms are dry eye, blurring of vision and foreign body sensation [16].

Approximately 40–45% of COVID-19-positive patients appear to be asymptomatic [17]. A cross-sectional study in China reported that 12.73% of patients presented with blurring of vision [15]. In our case, the patient complained of only blurring of vision and reported no other common ocular symptoms.

To our knowledge, there is only one case report of co-infection of COVID-19 and CMV, but the authors made no mention of any eye findings [18]. There are no reported cases of FBA in an immunocompromised patient with concurrent COVID-19 infection to date, which places us in a dilemma regarding the cause of this patient’s FBA. In a recent article, Le Balc’h *et al*. [19] reported that they detected frequent reactivation of CMV among patients diagnosed with COVID-19, but there was no ocular involvement mentioned in the study. Although serum CMV serology for our patient was positive, we should not rule out the possibility that concurrent COVID-19 infection might have aggravated the occurrence of FBA in this case.

Another case reported in Spain showed a possible association of retinal vasculitis with COVID-19 in children with chilblains [20]. Although the case was reported in children and not in adults, it did show that there might be some association between retinal vasculitis and COVID-19 regarding the cause of FBA.

The role of COVID-19 in ocular manifestation is still not well understood. This is the first reported case that describes the coexistence of FBA in a HIV-infected patient diagnosed with CMV and COVID-19 infection. Further research is needed to understand this condition better.

## Conclusion

Amidst the COVID-19 pandemic, clinicians should be aware of and take necessary preventive measures to be protected with appropriate personal protective equipment for possible coexistence of COVID-19 in any patient presenting with any eye symptoms, such as blurring of vision, eye redness, tearing, dry eye and foreign body sensation, despite the absence of clinical features suggestive of COVID-19. Whether FBA is one of the ocular signs of co-infection of COVID-19 and CMV remains unknown. Further studies are needed to provide more information on the ocular signs presenting in patients with concurrent COVID-19 and CMV infections.

## Data Availability

Not applicable.

## Abbreviations

AIDS: Acquired immunodeficiency syndrome
CMV: Cytomegalovirus
COVID-19: Coronavirus disease 2019
FBA: Frosted branch angiitis
HAART: Highly active antiretroviral therapy
HIV: Human immunodeficiency virus
IV: Intravenous
PCR: Polymerase chain reaction
